# Repurposing Normal Chromosomal Microarray Data to Harbor Genetic Insights into Congenital Heart Disease

**DOI:** 10.3390/biology12101290

**Published:** 2023-09-27

**Authors:** Nephi A. Walton, Hoang H. Nguyen, Sara S. Procknow, Darren Johnson, Alexander Anzelmi, Patrick Y. Jay

**Affiliations:** 1 Department of Pediatrics, Washington University School of Medicine, St. Louis, MO 63110, USA; 2 Department of Pediatrics, UT Southwestern Medical Center, Dallas, TX 75390, USA; hoang.nguyen@utsouthwestern.edu; 3 Genomic Medicine Institute, Geisinger, Danville, PA 17822, USA; 4 Department of Medicine, Thomas Jefferson University Hospitals, Philadelphia, PA 19107, USA

**Keywords:** chromosome microarray, congenital heart disease, data mining, genetic diagnosis, precision medicine, genetic testing, bioinformatics, functional genomics

## Abstract

**Simple Summary:**

About 15% of people born with congenital heart disease (CHD) have a specific genetic abnormality called a copy number variant. Most of their genetic tests, called chromosomal microarrays (CMAs), are considered normal. However, we suspected that some very small genetic deletions might be linked to CHD even though they were not reported in the test results. To investigate this, we investigated genetic test data from 319 patients with CHD. Then, we focused on genes in these small deletions that were somehow related to CHD, based on certain criteria like their association with CHD, their expression level in fetal hearts, and the potential impact of losing these genes. After analyzing the data, we found that these unreported small genetic deletions were slightly more likely to involve genes known to be related to CHD and also genes that might be important but were not recognized before. Our study suggests that “normal” genetic test data, which is readily available, can be valuable for discovering new genetic links to CHD. Also, smaller genetic deletions should be given more clinical attention for potential implications in CHD.

**Abstract:**

About 15% of congenital heart disease (CHD) patients have a known pathogenic copy number variant. The majority of their chromosomal microarray (CMA) tests are deemed normal. Diagnostic interpretation typically ignores microdeletions smaller than 100 kb. We hypothesized that unreported microdeletions are enriched for CHD genes. We analyzed “normal” CMAs of 1762 patients who were evaluated at a pediatric referral center, of which 319 (18%) had CHD. Using CMAs from monozygotic twins or replicates from the same individual, we established a size threshold based on probe count for the reproducible detection of small microdeletions. Genes in the microdeletions were sequentially filtered by their nominal association with a CHD diagnosis, the expression level in the fetal heart, and the deleteriousness of a loss-of-function mutation. The subsequent enrichment for CHD genes was assessed using the presence of known or potentially novel genes implicated by a large whole-exome sequencing study of CHD. The unreported microdeletions were modestly enriched for both known CHD genes and those of unknown significance identified using their de novo mutation in CHD patients. Our results show that readily available “normal” CMA data can be a fruitful resource for genetic discovery and that smaller deletions should receive more attention in clinical evaluation.

## 1. Introduction

A chromosomal microarray (CMA) test can return a genetic diagnosis in a substantial fraction of children who have a congenital malformation or neurodevelopmental disorder. For example, the diagnostic yield was between 9 and 20% in a large cohort of non-syndromic and syndromic congenital heart disease (CHD) patients who were evaluated in a clinical setting [1]. This means that most patients have normal CMA test results. While a negative result is not diagnostic, the raw CMA data could still be useful for scientific discovery. A pathogenic copy number variant (CNV) may go unrecognized because it is unknown or smaller than the typical 50 kb to 250 kb threshold for a microdeletion to be considered. The large amount of normal CMA data available at any major pediatric referral center could thus be a valuable resource for gene discovery if unreported pathogenic CNVs are sufficiently common.

Data mining offers appealing advantages over a prospective study but also certain challenges. The foremost advantage is the savings on recruiting, phenotyping, and testing patients. Pediatric specialists routinely evaluate patients, documenting their findings in clinical notes and imaging reports. In addition, sample sizes even from a single center could be quite large because only a fraction of eligible patients are ever recruited into a prospective research study. On the other hand, a prospective study can recruit normal controls for comparison. Depending upon the study design, the controls may be siblings, parents, or unrelated but matched individuals. Given that healthy individuals have no need for clinical evaluation, they would have no genomic data in an electronic medical record. Alternative solutions for controls, such as children who undergo genomic testing but do not have the disease in question, would be required.

There is a wealth of untapped scientific information stored in testing laboratories and hospitals. Electronic medical records (EMRs) contain valuable material that is often overlooked, and raw data from lab tests contain information with considerable value for medical research and patient diagnosis. These data can be accessed inexpensively and mined easily with the potential to generate new scientific knowledge or deliver patients a long-awaited diagnosis. In this study, we use the largely overlooked and discarded data from reportedly normal chromosomal microarrays (CMAs) to show that there is value in these untapped data stores. Copy number variants are very common in the human genome and are a significant cause of developmental delay, autism, epilepsy, congenital malformations, and congenital heart disease. It is standard practice to perform a chromosomal microarray analysis on patients with congenital heart disease. While the raw chromosomal microarray data contain on average 500 copy number variants, most laboratories only report deletions greater than 100 kb and duplications greater than 500 kb. However, it is logical to think that these unreported small-copy number variants can produce clinically significant phenotypes if they are in the region of a gene or a gene promoter that has not been previously described to cause congenital heart disease. We analyzed the raw data from previously collected chromosomal microarrays of patients with congenital heart disease and identified new copy number variants that may cause congenital heart disease.

## 2. Materials and Methods

### 2.1. Patient Population

To test the hypothesis that unreported microdeletions are enriched for known and novel CHD genes, we obtained raw data on putatively normal CMAs from 1762 pediatric patients who were evaluated at St. Louis Children’s Hospital between 1 January 2009 and 31 December 2014. The Washington University School of Medicine clinical cytogenomics laboratory deems a CMA “normal” based on the absence of deletions greater than 200 kb and duplications greater than 500 kb, with the exception of copy number variation in regions commonly associated with benign copy number polymorphisms in multiple independent studies. Deletions and duplications that are less than 1 Mb and do not involve known genes are also not reported. This conforms loosely to the less restrictive American College of Medical Genetics (ACMG) guidelines, which only state that a microarray analysis should detect duplications and deletions larger than 400 kb but does not specifically give size-based criteria for reporting [2] the laboratory-reported deletions greater than 200 kb and duplications greater than 500 kb. From 2009 to 2011, tests were performed using the Affymetrix SNP6.0 platform (Affymetrix, Santa Clara, CA, USA). From 2012 to 2014, tests were performed using the Affymetrix Cytoscan HD.

A pediatric cardiologist (H.H.N.) reviewed the medical records of all 1762 patients to ascertain their cardiac diagnoses, i.e., either normal or CHD. CHD diagnoses were further classified by 28 different cardiac phenotypes and groups. This review encompassed all available ambulatory clinic notes by cardiologists and clinical geneticists, echocardiogram and cardiac catheterization reports, and cardiac surgical operative notes. The demographic information collected was limited to gender and race. Patient demographics and CHD status are listed in Table 1. Table 2 lists phenotypes with the number of patients for each phenotype. Of the 1762 patients, 319 patients (18%) had a congenital heart defect.

### 2.2. Evaluation of a Probe-Number Threshold for the Detection of Microdeletions

Raw CMA data were processed using Nexus Copy Number Software (Biodiscovery Inc., El Segundo, CA, USA) to identify any deletion that was covered by at least three consecutive probes. The sequences were aligned to the GRCh37/hg19 reference genome. To determine the minimum number of probes necessary to detect a microdeletion reliably, we compared the CMAs from two pairs of monozygotic twins and two singletons whose tests were inadvertently performed twice. There were, in essence, four replicate pairs of CMAs. Each twin pair was tested concurrently using either the SNP6.0 or Cytoscan HD platform. Each singleton had two blood samples obtained 3–4 weeks apart, and the replicate test was performed using the same SNP6.0 or Cytoscan HD platform. The fraction of reproducibly detected microdeletions was calculated as a function of probe number. A microdeletion was included for subsequent analysis based on a 20-probe size threshold, as explained in the results.

### 2.3. Identification and Filtering for Enrichment of CHD Genes

We wrote an algorithm to count genes in microdeletions and to compare counts between cases and controls. Both populations in this study had a clinical indication for CMA testing, but the controls had no congenital heart defect. A gene deletion was counted if at least one exon of the gene was within a microdeletion. Comparisons between cases and controls were performed for each of the 28 cardiac phenotypes or phenotype groups and 17,491 autosomal genes to calculate an odds ratio and nominal *p*-value using a Fisher’s exact test (N = 489,748 comparisons). A nominally significant set of genes was defined from the comparisons. To be included in the set, a gene had to have a deletion in at least two cases and less than 5% of controls, and a deletion had to increase the risk of a CHD phenotype with an odds ratio ≥ 1.5 and a nominal *p*-value < 0.05.

**Figure 1:**
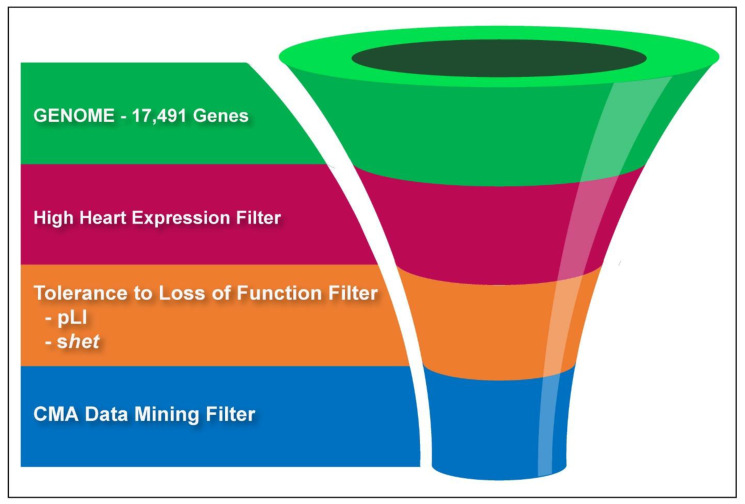
Gene filtering process.

To enrich for CHD genes, the nominally significant gene set was sequentially filtered by one or two bioinformatics criteria. First, we selected genes in the top quartile of expression in the fetal mouse heart, as previously described by [4]. Genes that lacked expression information were retained as well. Second, we filtered genes using their pLI or *s*_het_ score, two different quantitative estimates for the deleteriousness of a loss-of-function mutation based on the Exome Aggregation Consortium (ExAC) data [5,6]. We selected genes with pLI > 0.9 or *s*_het_ > 0.1; the two thresholds are known to enrich for genes associated with diseases resulting from haploinsufficiency [5,6]. A graphical illustration of our filtering process is shown in Figure 1.

We estimated the significance of CHD gene enrichment using a test of two proportions with a two-tailed Z-test [7]. To curate the known CHD gene set, we searched the literature published as of 31 December 2016 (Appendix A). A mutated gene was determined to cause human CHD according to one of three requirements: (1) Mutations of the gene caused a well-described syndrome that involves CHD, and at least one patient was shown to have the mutation. (2) Mutations of the gene were established as a cause with significant association in a family or unrelated cases. (3) A mutation was described in only one or two cases, but a mutant animal model demonstrated a CHD phenotype.

To evaluate the potential of our approach to yield novel CHD genes, we quantified the enrichment for genes associated with de novo mutation in a large whole-exome sequencing study compared to the enrichment in controls used in that same study. The study performed by Sifrim et al. identified several genes that met statistical criteria to conclude a pathogenic role. The vast majority of the hundreds of genes identified in the Sifrim study that did not achieve statistical significance were presumably enriched for novel CHD genes [8]. All the mutated genes were listed in the supplementary data as two datasets, de novo variants found in syndromic cases of CHD (Supplementary Table S21 in ref. [8]) and de novo variants found in non-syndromic cases of CHD (Supplementary Table S22 in ref. [8]). The Sifrim study used de novo variants from two control populations without CHD as controls. The first included de novo variants from a cohort of 900 trios with exome sequencing from the Simons Foundation Autism Research Initiative Simplex Collection [9], with each trio consisting of the unaffected parents and a sibling of a child with autism spectrum disorder. The second set was derived from the Deciphering Developmental Disorders Study, using cases from that study that did not have CHD [10]. For our study, we excluded the controls derived from the Deciphering Developmental Disorders study population due to the strong relationship between CHD and other developmental disorders, particularly in the context of copy-number variation. The significance of novel CHD gene enrichment was evaluated using a test of two proportions, as described above, using the Sifrim syndromic and non-syndromic gene sets. For comparison, we evaluated the enrichment in the control population of de novo mutations in unaffected siblings of children with autism spectrum disorder. Genes that overlapped between cases and controls were excluded from the analyses.

## 3. Results

### 3.1. A CMA Probe-Number Threshold Reliably Detects Microdeletions Smaller Than 200 kb

**Figure 2:**
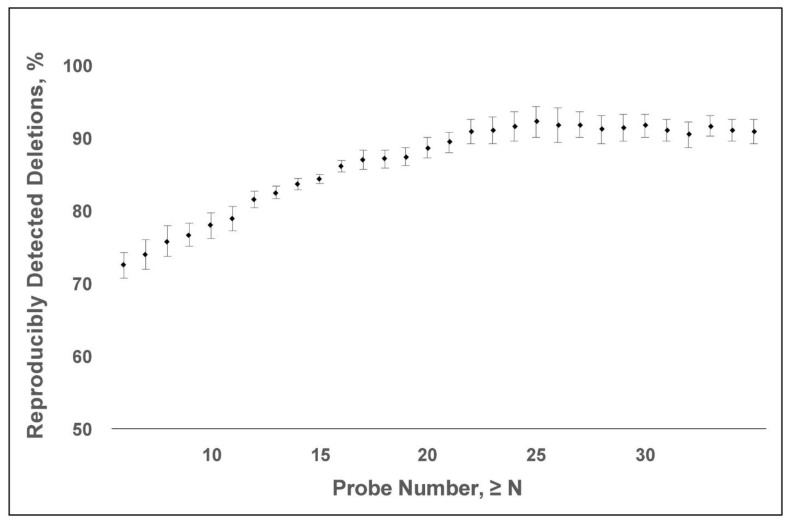
The number of deletions that were reproducible between identical twins (*n* = 2) and between independently replicated CMAs from the same patient (*n* = 2) and based on the minimum number of probes used to call the deletion. A minimum probe threshold of 20 probes provides an approximately 90% positive predictive value for a deletion being reproducible.

**Figure 3:**
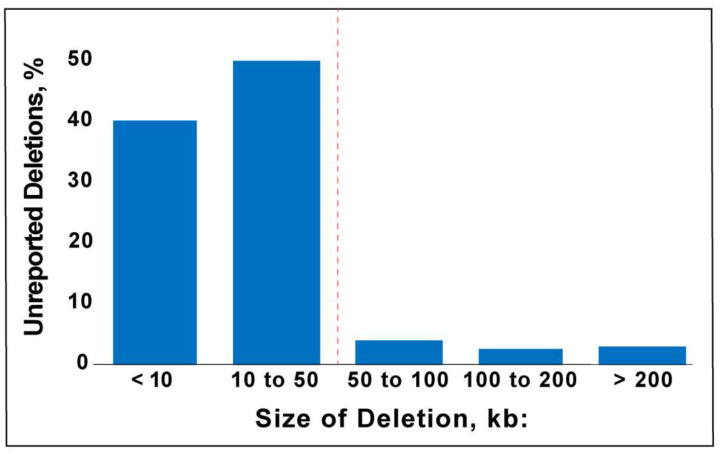
Size distribution of unreported deletions.

Studies of healthy individuals indicate that microdeletions <500 kb are largely benign [11,12,13]. The empirical findings support the typical <200 kb threshold for reporting a microdeletion in the diagnostic interpretation of a CMA. In contrast, the detection of a CNV on an oligonucleotide microarray depends on the SNP probe coverage. Given that the density of SNP probes varies across the genome, a CMA-probe number threshold could enable the detection of <200 kb microdeletions and the re-purposing of putatively normal CMA data for gene discovery. We calculated the reproducibility of detecting microdeletions as defined by probe number using four pairs of “replicate” CMAs. The CMAs were obtained from two monozygotic twin pairs and two individuals who had two replicate tests. A 20-probe threshold has ~90% positive predictive value for microdeletion (Figure 2). Based on this probe-number threshold, the average size of a microdeletion is 47 kb *±* 244 kb (S.D.), with a median size of 13 kb. Overall, 97% of the deletions were below the 200 kb reporting threshold and 90% were below 50 kb, which was the lowest reporting threshold of all the labs we assessed (Figure 3). Deletions greater than 200 kb are not reported when they are present in areas of common variation where deletions are common and thought to be benign or in regions where there are no known genes.

### 3.2. Unreported Microdeletions in CHD Patients Are Enriched for Known CHD Genes

Unreported microdeletions may cause haploinsufficiency of a CHD gene. We quantified the incidence of this possibility in the 319 CHD and 1400 control patients who had putatively normal CMAs from 2009 to 2014 at our institution. We assessed case and control populations for deletions of the 171 known human CHD genes as of 31 December 2016 (Appendix A) and found that 15% (48/319) of patients with CHD had haploinsufficiency in at least one of these genes compared with 9.5% (168/1762) (*p* = 0.005) of patients without CHD. Mutations that perturb the development of multiple organs, such as the heart and brain, likely contribute to the low incidence of CHD gene deletion in non-CHD patients [8,14].

### 3.3. Sequential Filtering of Normal CMA Data Enriches for Known CHD Genes

Our list of known CHD genes represents less than 1% (0.937%) of the total genes in our analysis. If we filter this complete list of genes by selecting only those genes where cases (i.e., cardiac phenotype groups) had significantly more deletions than controls (*p* < 0.05), we expected enrichment of CHD genes if these associations are meaningful. Because benign CNVs far outnumber pathogenic ones, we expected that many of the genes detected in our analysis were false positives and may even neighbor CHD genes in some deletions. We thus sought to enhance the yield of mining normal CMA data by filtering genes using a series of additional criteria to enhance the probability that we selected genes known to cause CHD. With each additional filter, we assessed the enrichment for known CHD genes using a comparison to the previously filtered set. Our filtering process is outlined in Figure 1.

**Figure 4:**
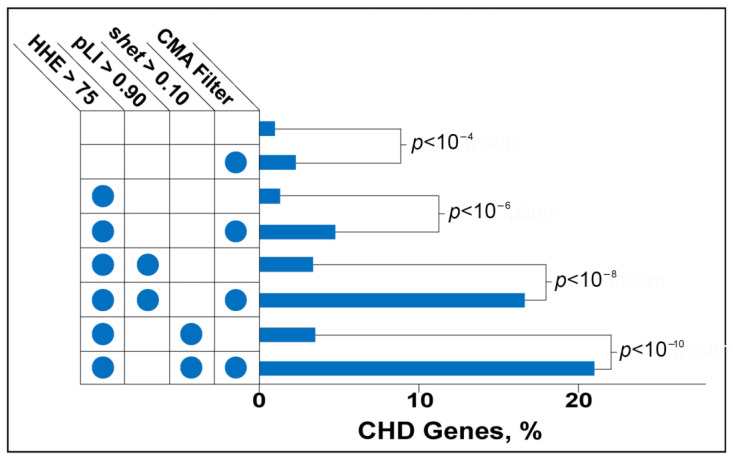
Enrichment of CHD genes based on the filters applied (indicated with dots on the left). Our CMA algorithm filter enriched for CHD genes in every combination with the highest amount of enrichment when combined with filters for the heterozygous loss of function score and fetal heart expression achieving a greater than 21-fold enrichment in CHD genes.

After applying the first filter selecting only genes that were deleted more frequently in patients from any CHD phenotype group compared with controls, we yielded a significant, 2.33-fold enrichment (*p* < 0.0004) for known CHD genes (Figure 4).

The second filter selected genes that are highly expressed in the fetal mouse heart. Zaidi et al. first applied the “high heart expression” (HHE) criterion to a large set of de novo mutations found in CHD patients. The filter helped to hone in on the critical role of epigenetic regulators in CHD [4]. When applied to the CMA-filtered gene set, the HHE filter eliminated 60% of the non-CHD genes. Non-CHD genes appeared to be selectively eliminated because the combination of CMA and HHE filters increased the absolute enrichment for known CHD genes to 4.78% and the relative enrichment to 2.33%, representing a (4.87)-fold compared to no filters (Figure 4).

The third filter selected genes for which the loss-of-function was predicted to be deleterious. We used two versions of the filter with either the pLI or s_het_ score of a gene. The two metrics derive from different statistical models that estimate deleteriousness from ExAC data [5,6]. One-third of the 3230 genes that have a pLI score > 0.9 are associated with diseases of haploinsufficiency. The other two-thirds have no known disease phenotype yet [5]. As expected, genes filtered by high heart expression and a pLI > 0.9 are enriched for CHD genes compared with the baseline (3.39% versus 0.98%). Applying the CMA filter further increases CHD-gene enrichment by nearly 3.5-fold (3.49). CMA, HHE, and pLI filtering resulted in a set of 72 genes; 16.66% are known CHD genes (Figure 4). Of the 2984 genes that have s_het_ score > 0.1, more than 20% are associated with the autosomal dominant disease. CHD genes have relatively high s_het_ scores, and genes for congenital disorders, in general, are strongly associated with s_het_ > 0.1 [6]. Also, as expected, genes that show high heart expression and have s_het_ > 0.1 are enriched for CHD genes compared with the baseline, (3.53% versus 1%). Notably, the combination of CMA, HHE, and s_het_ > 0.1 filters yields the greatest enrichment for CHD genes. The resulting set includes 57 genes that show a 21-fold enrichment for CHD genes relative to no filter. Known CHD genes comprise 21% of the set (Figure 4).

The correlation between pLI and s_het_ scores is strong but not perfect, resulting in similar but not identical gene sets [15]. Each set contains 26 and 11 unique genes, respectively, and 58 overlapping genes for a total of 95 genes (Appendix B). The two sets contain 12 CHD genes that were known as of 31 December 2016. An additional gene, *SKI*, was missed in our initial assessment of known CHD genes. Before publication, several authors moved to different institutions, leading to a delay in the release of this study. Consequently, the 95 genes identified in the research were re-evaluated six years after the study’s conclusion using a literature review to ascertain their relevance in the pathogenesis of CHD. Since the completion of this study, mutations of *ABL1*, *CELSR1*, *DST*, *PRPF8*, *CTBP1*, *ATP6V1E1*, and *USP34* have been associated with human CHD, and *PTEN* has been implicated [16,17,18]. Thus, approximately 22% of the 95 genes that were identified using sequential filtering of normal CMA data have well-documented or compelling evidence for their role in human CHD.

### 3.4. Enrichment for Novel CHD Genes Using Sequential Filtering of Normal CMA Data

The enrichment for known CHD genes suggests the potential of using normal CMA data for gene discovery. To estimate the yield of novel genes, we compared the overlap between the two gene sets resulting from CMA, HHE, and pLI or s_het_ filtering and the genes found in a large whole-exome sequencing study by Sifrim and colleagues. This study found that syndromic CHD cases are strongly associated with de novo, loss-of-function mutations, as compared with unaffected controls or non-syndromic CHD cases. Sifrim et al. classified CHD cases by syndromic CHD (S-CHD) or non-syndromic CHD (NS-CHD) status. They identified de novo mutations of 754 and 1060 genes in 409 syndromic and 561 non-syndromic cases. Despite the large sample size, fewer than 2% of the genes in the Sifrim study achieved the statistical significance necessary to conclude a pathogenic role. It is likely, however, that the remaining 98% that did not achieve statistical significance are enriched for novel CHD genes. We therefore quantified the overlap between our filtered gene sets and the Sifrim dataset. A greater-than-expected overlap was considered to be consistent with an enrichment for novel CHD genes.

**Figure 5:**
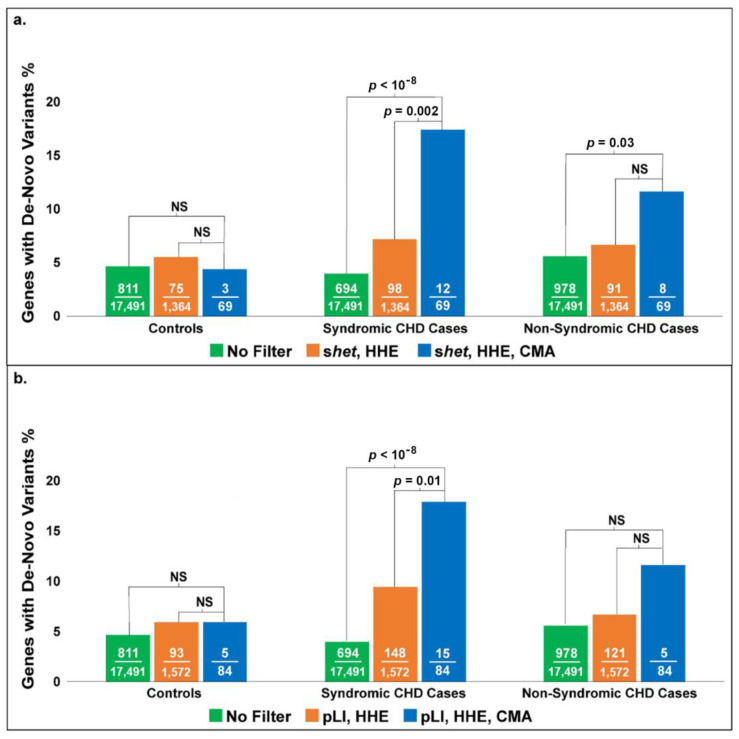
The two graphs show the enrichment of genes with de novo mutations in exome studies of CHD patients and controls using different filtering methods (S_het_ (**a**) versus pLI (**b**)).

When we evaluated our dataset for the enrichment of genes with de novo mutations from the Sifrim study, we achieved significant enrichment of genes in the S-CHD patients from the Sifrim dataset using both pLI filters (*p* < 10^−8^) and *s*_het_ (*p* < 10^−8^), as shown in Figure 5a,b. There was no significant enrichment in the NS-CHD patients using the pLI filter, but there was significant enrichment using the *s*_het_ filter (*p* = 0.03) (Figure 5a); however, this was not as significant as the enrichment for S-CHD patients. Genes within the control patient population who had de novo variants but no CHD did not show any enrichment in our study, further validating our results. These findings are consistent with previously published studies on the genetics of CHD. The genetic etiology of NS-CHD has been very hard to determine. We expected that our model would show more enrichment with S-CHD as deletions more often cause S-CHD.

Our two models produced a combined list of 95 genes. In total, 12 of these genes represented known CHD genes from our list. A list of these genes is included in Appendix B.

## 4. Discussion

Our two models produced a combined list of 95 genes (Appendix B). In total, 13 of these genes represented known CHD genes. Although variants in these genes are known to cause CHD, it is notable that deletions have not been described as causing CHD in most of these genes. Seven genes from our list, *ABL1*, *CELSR1*, *DST*, *PRPF8*, *CTBP1*, *ATP6V1E1*, and *USP34*, were described to cause CHD since our initial review and analysis. Excluding the known and recently discovered genes, we identified 82 novel candidate genes for CHD. Thirteen of these genes are deleted in combination with another candidate gene in the list. For example, *DAZAP1*, *RPS15*, and *MBD3* all lie on chromosome 19 and are deleted together in most cases. *DAZAP1* and *MBD3* do not currently show any evidence for their involvement in CHD; however, a missense mutation in *RPS15* has been described as a possible causal candidate in a patient with complex CHD as part of Diamond Blackfan anemia [19]. Two of the candidate genes were determined to be passenger genes, in that they were deleted in combination with a known CHD gene. *SEPT5* and *UBE2I* were passengers of known CHD genes *TBX1* and *IFT140*, respectively. Neither of these known genes showed up in our final list because they did not meet the filtering threshold. *TBX1* did not meet the filtering threshold for fetal heart expression and *IFT140* did not meet the threshold for HHE, *s*_het_ , or pLI score. None of the passenger genes were found to overlap with genes with variants found in CHD exome studies, whereas five out of the seven genes that were discovered to be CHD genes after our review were also found in CHD exome studies. In total, 25 (~30%) of our candidate genes are genes that were found to have de novo mutations in CHD exome studies. Five of these have since been described to cause CHD and another five were shown to cause CHD in animal models. As 40% of the overlapping genes show significant evidence supporting their role in CHD, we consider the remaining 15 overlapping genes to be high-probability candidates that merit further study (Table 3).

*TBX1* is perhaps the most well-known gene where a deletion is known to cause CHD. *TBX1* did not show up in our final filter because it did not meet the threshold for fetal heart expression (12 vs. min 75). Despite this, the gene has a high pLI of 0.98, a high *s*_het_ score of 0.231, and with the phenotype of VSD, reached the level of statistical significance even after Bonferroni correction (odds ratio 4.12, *p*-value 9.82 *×* 10^−14^). Using a sliding scale, we can adjust these values and perhaps pick up more candidate genes for CHD. The fact that this common cause of CHD shows up with such high significance in our study suggests that we are missing known deletions that cause disease and underscores the importance of reconsidering our methodology for calling CNVs from CMA, as these are all CMAs that were reported as “normal”. Other methodologies, that are able to detect smaller CNVs may be important for further diagnostic workup of CHD.

Our analysis showed that there is valuable information in unreported chromosome microarrays that we believe may have implications for scientific research and clinical care of patients. First, there is currently an abyss between CMA results and exome analysis, where deletions smaller than 100 kb are often overlooked. Providing better testing coverage for these smaller copy number variants could provide a diagnosis for a considerable number of patients. This could be addressed by reducing the threshold we use to call CNVs on CMA or by improving sequencing platforms to call CNVs with more accuracy from exome or whole genome data. Notably, there have been significant improvements in probe density on CMA platforms without significant changes in our threshold for calling CNVs. Large patient cohorts with available CMA data likely contain significant numbers of “knock-out” humans whose gene deletions combined with clinical phenotype data could provide substantial information about gene function and human disease. From a research perspective, this work shows the potential for largely unused data that sit in our laboratories to be mined for gene discovery. Filtering resulting gene sets from analyses of this information using data from various gene function studies has the potential to produce high-probability candidate genes for further study in animal models or to be further assessed for testing in humans.

## 5. Limitations

Our study was constrained by the limited number of chromosome microarrays available, and this limitation was further compounded by the subset of those patients diagnosed with CHD. Despite the modest sample size, we believe that we showcased significant value even within this restricted dataset. We also recognize the limited number of samples utilized to establish the probe number threshold for detecting deletions. This constraint arose from the few patients with duplicate microarrays. Nonetheless, we deemed this metric preferable to seemingly arbitrary thresholds identified in other studies.

## 6. Conclusion

In summary, we conclude that patients with “normal” CMAs may have overlooked clinically relevant pathogenic deletions. Raw CMA data combined with phenotypic information can be mined for gene discovery. Other forms of “normal” genomic data can be similarly mined for gene discovery by combining other gene information such as pLI score, gene expression, and *s*_het_ to help find candidate genes in the face of difficulty in obtaining genome-wide significance. Small deletions are an overlooked and major cause of human genetic disease that need more attention in the clinical space.

## Figures and Tables

**Table 1:** Patient demographics. Between 1 January 2009 and 31 December 2014, 1782 patients evaluated at St. Louis Children’s Hospital had a normal CMA test. The preponderance of males among the non-CHD patients reflects the male predominance in autism, for which a CMA is commonly ordered. The slightly higher fraction of males among CHD patients is consistent with epidemiological data [3]. The gender ratios between CHD and non-CHD groups are significantly different (*p* < 0.01, two-tailed chi-squared test). The racial composition of CHD and non-CHD patients is similar and representative of the region surrounding St. Louis Children’s Hospital, a major referral center.

	CHD	(Number)	Non-CHD	(Number)
	18%	319	82%	1443
**Gender**				
Female	48%	152	39%	569
Male	52%	167	61%	874
**Race**				
White	75%	240	77%	1116
Black	14%	45	15%	218
Other	11%	34	8%	109

**Table 2:** Cardiac phenotypes with the number of patients affected. There is some overlap between patients with phenotypes where a given patient may have exhibited more than one phenotype.

Phenotype Group	Cardiac Phenotype	No. of Patients
**All Congenital Heart Disease**		**319**
**Conotruncal/Anterior Second Heart Field Defects**		**46**
	Tetralogy of Fallot	25
	Truncus Arteriosus	3
	AP Window	1
	Interrupted Aortic Arch Type B	1
**Left-Sided Obstructive Lesions**		**79**
	Hypoplastic Left Heart Syndrome	28
	Coarctation of Aorta	34
	Aortic Stenosis	4
	Bicuspid Aortic Valve	22
**Simple Septal Defects**		**125**
	All Atrial Septal Defects (Including Resolved)	51
	Repaired Atrial Septal Defect	29
	Ventricular Septal Defect	89
**Other Heart Defects**		
	Double Outlet Right Ventricle	27
	Dextro-Transposition of the Great Arteries	23
	Pulmonary Atresia	24
	Heterotaxy	16
	AV Canal	22
	Tricuspid Atresia	14
	Interrupted Aortic Arch	6
	Double Inlet Left Ventricle	6
	Ebstein’s Anomaly	4
	Pulmonic Stenosis	4
	Cardiomyopathy	10
	TAPVR	8
	Pulmonary Vein Stenosis	3

**Table 3:** Candidate genes overlapping between this study and genes with de novo variants in the Sifrim et al. and Homsy et al. exome studies. Genes *CTBP1* [20] and *ATP6V1E1* [21] were also found to cause CHD but did not overlap with the exome studies.

Recently Discovered	Evidence in Animal Models	High Probability Candidate Genes		
*ABL1* [16,22]	*ARHGDIA* [23]	*AGPAT3*	*CEP170B*	*SEMA4D*
*CELSR1* [24,25]	*ERBB2* [26,27,28]	*AHDC1*	*CYFIP1*	*SMG6*
*DST* [29]	*IGF2R* [30]	*ARCN1*	*PAFAH1B1*	*SYMPK*
*PRPF8* [31]	*SMARCC1* [32]	*BIRC6*	*PTPRD*	*SYNGAP1*
*USP34* [17]	*TNS1* [33]	*BRD4*	*PUM1*	*WSB1*

## Data Availability

The datasets generated and/or analyzed during the current study are not publicly available due to the privacy of patient genetic information. The resulting gene list is provided in Appendix B.

## Appendix A. Known CHD Genes

**Known CHD Genes**								
*ACTB*	*CCDC151*	*DNAAF3*	*FGFR1*	*KANSL1*	*MYH7*	*PKD1L1*	*SEMA3E*	*TBX1*
*ACTC1*	*CCDC39*	*DNAH5*	*FIG4*	*KAT6B*	*NEK1*	*PRKD1*	*SETBP1*	*TBX20*
*ACVR2B*	*CCDC40*	*DNAI1*	*FOXC1*	*KCNJ2*	*NEK8*	*PTPN11*	*SF3B4*	*TBX3*
*ADAMTS10*	*CDC45*	*DNAI2*	*FOXC2*	*KMT2D*	*NF1*	*PUF60*	*SH3PXD2B*	*TBX5*
*ADNP*	*CDK13*	*DNAL1*	*FOXF1*	*KRAS*	*NIPBL*	*RAB23*	*SHH*	*TCOF1*
*AFF4*	*CDKN1C*	*DOCK6*	*FOXH1*	*LEFTY2*	*NKX2-5*	*RAD21*	*SHOC2*	*TDGF1*
*ANKRD11*	*CEP57*	*DYNC2H1*	*G6PC3*	*LMBRD1*	*NKX2-6*	*RAF1*	*SMAD3*	*TEK*
*ANKS6*	*CFC1*	*DYX1C1*	*GATA4*	*LTBP2*	*NME8*	*RARB*	*SMAD4*	*TFAP2B*
*ARHGAP31*	*CHD4*	*ECE1*	*GATA6*	*LTBP4*	*NODAL*	*RBM8A*	*SMAD6*	*TKT*
*ARID1A*	*CHD7*	*EFTUD2*	*GBA*	*MAP2K1*	*NOTCH1*	*RIT1*	*SMARCA2*	*TLL1*
*ARMC4*	*CHST14*	*EHMT1*	*GDF1*	*MAP2K2*	*NOTCH2*	*ROR2*	*SMARCA4*	*TRAP1*
*B3GAT3*	*CITED2*	*ELN*	*GJA1*	*MED13L*	*NPHP3*	*RPL11*	*SMARCB1*	*TRIM32*
*BBS10*	*CREBBP*	*EOGT*	*HOXA1*	*MEGF8*	*NPHP4*	*RPL35A*	*SMARCE1*	*TTC37*
*BBS2*	*CRELD1*	*EP300*	*HRAS*	*MEIS2*	*NR2F2*	*RPL5*	*SMC3*	*TTC8*
*BRAF*	*DDX11*	*ERBB3*	*IFT140*	*MGP*	*NRAS*	*RPS19*	*SMG9*	*WDPCP*
*CACNA1C*	*DHCR7*	*ESCO2*	*IFT172*	*MKKS*	*NSD1*	*RPS24*	*SNRPB*	*WDR35*
*CBL*	*DLL4*	*EVC*	*INVS*	*MKS1*	*PDGFRA*	*RSPH4A*	*SOS1*	*ZEB2*
*CCDC103*	*DNAAF1*	*EVC2*	*IRX5*	*MMP21*	*PIGL*	*SALL1*	*STRA6*	*ZFP57*
*CCDC114*	*DNAAF2*	*FGF8*	*JAG1*	*MYH6*	*PITX2*	*SALL4*	*TAB2*	*ZFPM2*

## Appendix B. Filtered Gene List

**Combined Genes from Filters**							
*ABL1*	*BIRC6*	*DCTN2*	*HNRNPR*	*OGDH*	*PTPRD*	***SKI***	*TOPORS*
*AGPAT3*	*BRD4*	*DST*	*IGF2R*	*OR13C5*	*PUM1*	***SMARCA4***	*UBAP2*
*AHDC1*	*CAPN15*	*EDF1*	*IL17REL*	*P4HB*	*RABL6*	*SMARCC1*	*UBE2I*
*ALYREF*	*CELSR1*	***EHMT1***	***KANSL1***	*PAFAH1B1*	***RAF1***	***SMC3***	*UBE3A*
*ANAPC2*	*CEP170B*	***EP300***	*KANSL2*	*PDIA3*	*RAI1*	*SMG6*	*UBQLN1*
*ANKFY1*	***CHD7***	*ERBB2*	*KCNH2*	*PGP*	*RAPGEF1*	*SUMO2*	*USP34*
*APC*	*COPS3*	*FASN*	*MBD3*	*PIP4K2B*	***RBM8A***	*SYMPK*	*WDR18*
*ARCN1*	*CRTC1*	*FBXO11*	*NAP1L4*	*PRPF8*	*RNPS1*	*SYNGAP1*	*WSB1*
*ARHGDIA*	*CTBP1*	***FGFR1***	*NF2*	*PSMB2*	*RPS15*	***TFAP2B***	*YWHAE*
*ARNT*	*CUX1*	*GSK3A*	***NOTCH1***	*PTBP1*	*SEMA4D*	*TFG*	*ZC3H18*
*ATP6V1E1*	*CYFIP1*	*GTPBP1*	*NPLOC4*	*PTEN*	*SEPT5*	*TM9SF4*	*ZMIZ2*
*ATXN2*	*DAZAP1*	*HERC2*	*NUP85*	***PTPN11***	*SET*	*TNS1*	
(Bolded genes are known CHD genes).							
